# Inequalities in the assessment of childhood short stature

**DOI:** 10.3399/bjgp23X732309

**Published:** 2023-03-31

**Authors:** Justin H Davies, Jenny Child, Joseph Freer, Helen L Storr

**Affiliations:** Regional Centre for Paediatric Endocrinology, Southampton Children’s Hospital, Faculty of Medicine, University of Southampton, Southampton; Co-Chair, British Society of Paediatric Endocrinology Growth Disorders Special Interest group (BSPED GD-SIG).; Parent of a child with a growth disorder; member, BSPED GD-SIG; Global Registry For Novel Therapies In Rare Bone & Endocrine Conditions (GloBE-REG); Patient Advisor, Genomics England.; Wolfson Institute of Population Health, Queen Mary University of London, London.; Centre for Endocrinology, William Harvey Research Institute, Barts and the London School of Medicine, Queen Mary University of London, London; Chair, BSPED GD-SIG.

## WHY IS ASSESSMENT OF CHILDHOOD SHORT STATURE IMPORTANT?

Measurement of height is a key part of the assessment of a child’s health. Short stature is pragmatically defined as height less than the second centile, plotted on sex-specific growth charts. Although the commonest cause of short stature is familial, it may be the earliest and/or only presenting feature of serious childhood illness and therefore requires investigation (see Clinical Practice article in this *BJGP* Issue).^1^ In some cases, short stature is a marker of socioeconomic vulnerability; in England it strongly tracks to areas with higher levels of deprivation.^2^ Early detection of short stature is key to optimising health outcomes. Delayed diagnosis can result in reduced adult height and failure to address the underlying pathology can have potentially serious consequences. Short stature in young children may also be an important predictor of future cognition.^3^

Short stature is often associated with significant child and caregiver anxiety. The child may experience low self-esteem, anxiety, and bullying at school, exacerbating the situation:

*‘Tragically, as an almost 7-year-old, he looked 4 years of age. Our son was facing bullying, name calling, jeering and regularly being laughed at which made us start the growth investigation journey again.’*

(Suma, carer)

Caregivers often report feeling something has been missed and their concerns are not taken seriously:

*‘If one more midwife, GP, nurse, paediatrician (etc) said “you aren’t very tall” I would have screamed. I’m slim, but 50–75th centile for height and my son wasn’t on the charts. I felt patronised … ’*

(Jayne, carer)

*‘We were told, “It’s just a cosmetic issue!”’*

(Sara, carer)

The key clinical task in primary care is to distinguish those children with normal variant short stature from those requiring investigation and referral, while remaining cognisant of the child and family’s concerns.

There are significant social disparities in the assessment of childhood short stature. This presents additional challenges and it is crucial that clinicians recognise issues relating to gender, race, and poverty to improve timely diagnosis and reduce health inequalities.

## HEIGHT AS A MARKER OF SOCIAL INEQUALITY

Inequalities in height exist between countries, for example, mean height differences of up to 20 cm has been observed between the tallest and the shortest populations internationally.^4^ Differences also exist within countries. Data from the National Child Measurement Programme in England found a fourfold difference in the prevalence of short stature between the highest and lowest prevalence areas and observed that short stature was highly related to area-level deprivation, ethnicity, and sex.^2^

## INEQUALITIES IN THE ASSESSMENT OF CHILDHOOD SHORT STATURE

The initial assessment of short stature is typically performed in primary care following self-referral by the family. Inequalities in the assessment and management of short stature can be divided into factors relating to the caregiver, primary care, and specialist treatment.

Personal caregiver attitudes and societal pressures determine thresholds of concern regarding a child’s height. Men and women can have different perceptions of the impact of short stature in adulthood.^5^^,^^6^ Short stature is often considered less of a problem in women. So-called ‘heightism’ is generally a male phenomenon, leading to low self-esteem and creating barriers to success.^7^ Evidence from the US suggests that ‘thresholds of concern’ with respect to short stature vary between racial groups. Qualitative data suggests this may relate to poverty and access to care.^7^^,^^8^ Short stature may go unrecognised in communities where short children are more common, particularly where the child’s parents also experienced childhood short stature.

The gender, racial, and social disparities in the investigation and treatment of childhood short stature also exist in primary care, leading to disparate delays in referral and/ or diagnosis.

Girls are less frequently referred from primary care for assessment of short stature, yet often have more severe growth failure at presentation and have higher rates of underlying pathology compared with boys:^9^

*‘We were fobbed off for years, told she will grow and one doctor said “what do you want, invasive leg extension surgery?” At aged 14 years she was diagnosed with* [a treatable genetic cause] *but by then it was too late for her to have growth hormone which could have helped her so much.’*

(Nicky, carer)

*‘I was told so many times that “he would have a growth spurt” but it never came.’*

(Emma, carer)

*‘His mother has insisted on the referral; however, we are not concerned about growth currently. He was then diagnosed with GH* [growth hormone] *deficiency … ‘*

(Pauline, carer)

Bias inherent in health professionals also contributes to disparities in the investigation and treatment of short stature. White children, especially those from higher socioeconomic groups, are not only more frequently referred for concerns about growth but are also more likely to be offered growth hormone (GH) stimulation testing than children of other ethnicities.^9^ Black children are shorter at the time of referral and, in a study in Philadelphia, US, had lower GH levels during GH stimulation testing compared with White children, reflecting racial delays in referral and investigation.^10^ The degree of short stature determined the likelihood of receiving GH therapy in girls but not in boys, implying a sex bias in the GH treatment decision making. These biases seem to originate from children, parents, and clinicians.^11^^,^^12^ Black children and girls of all ethnicities are underrepresented in children receiving GH therapy. In the UK, twice as many boys receive GH for short stature compared to girls.^13^

Although the assessment and treatment of short stature should be driven by clinical concern, biased clinical decision making is disadvantaging girls and some ethnic minority groups, especially Black children. Paradoxically, these groups have the greatest need.

These systematic racial and gender biases in clinicians and families are often unconscious, but must be acknowledged and tackled before healthcare inequalities can be addressed.

## ADDRESSING INEQUALITIES IN THE ASSESSMENT OF CHILDHOOD SHORT STATURE

Raising awareness of the health disparities in the assessment of childhood short stature in caregivers, primary, secondary, and specialist clinicians is key to addressing these issues. Caregivers must be empowered to raise concerns about their child’s growth. There should be equal access to accurate auxological assessment and culturally appropriate educational materials. Clinicians should recognise their own implicit biases and reflect on how these may impact their management of short children. Paediatricians should ensure equal access to appropriate testing and GH therapy, in particular for girls and children from ethnic minority groups. Electronic algorithms or apps that interpret height measurements can help to standardise childhood growth assessments and reduce the biased clinical decision making:

*‘The diagnosis has had a huge positive impact on our daily lives, as all the unanswered questions and uncertainty has been lifted. Having this diagnosis will enable my daughter to get the help she so needs and deserves in school.’*

(Suma, carer)
